# A New Device for Step-Down Inhibitory Avoidance Task—Effects of Low and High Frequency in a Novel Device for Passive Inhibitory Avoidance Task That Avoids Bioimpedance Variations

**DOI:** 10.1371/journal.pone.0116000

**Published:** 2015-02-23

**Authors:** Gilvan Luiz Borba Filho, Kamila Cagliari Zenki, Eduardo Kalinine, Suelen Baggio, Letícia Pettenuzzo, Eduardo Rigon Zimmer, Simone Nardin Weis, Maria Elisa Calcagnotto, Diogo Onofre de Souza

**Affiliations:** 1 Programa de Pós-Graduação em Educação em Ciências, ICBS—Universidade Federal do Rio Grande do Sul, Porto Alegre, Brasil; 2 Programa de Pós-Graduação em Ciências Biológicas-Bioquímica, ICBS—Universidade Federal do Rio Grande do Sul, Porto Alegre, Brasil; 3 Departamento de Bioquímica, ICBS—Universidade Federal do Rio Grande do Sul, Porto Alegre, Brasil; 4 Programa de Pós-Graduação em Ciências Fisiológicas—Universidade Federal de Sergipe, São Cristóvão, Sergipe, Brasil; Université Pierre et Marie Curie, FRANCE

## Abstract

**Background:**

Step-down inhibitory avoidance task has been widely used to evaluate aversive memory, but crucial parameters inherent to traditional devices that may influence the behavior analysis (as stimulus frequency, animal’s bioimpedance) are frequently neglected.

**New Method:**

We developed a new device for step-down inhibitory avoidance task by modifying the shape and distribution of the stainless steel bars in the box floor where the stimuli are applied. The bars are 2mm wide, with rectangular shape, arranged in pairs at intervals of 1cm from the next pairs. Each pair makes an electrical dipole where the polarity inverts after each pulse. This device also presents a component that acquires and records the exact current received by the animal foot and precisely controls the frequency of stimulus applied during the entire experiment.

**Result:**

Different from conventional devices, this new apparatus increases the contact surface with bars and animal´s paws, allowing the electric current pass through the animal´s paws only, drastically reducing the influence of animal’s bioimpedance. The analysis of recorded data showed that the current received by the animal was practically the same as applied, independent of the animal´s body composition. Importantly, the aversive memory was observed at specific stimuli intensity and frequency (0.35 or 0.5 mA at 62 and 125Hz but not at 0.20 mA or 20 Hz). Moreover, with this device it was possible to observe the well-known step-down inhibitory avoidance task memory impairment induced by guanosine.

**Conclusion:**

This new device offers a substantial improvement for behavioral analysis in step-down inhibitory avoidance task and allows us to precisely compare data from different animals with distinct body composition.

## Introduction

In the last century many apparatus and protocols to study learning and memory have been created. One of these devices developed by Thorndike [1] consisted of small crates or ‘problem boxes’. In the late 1930’s, the task was performed with a box containing a lever and food supplier. In this device, the rat had to learn how to have access to the food by pressing the lever[2]. Later, Hudson and colleagues[3]presented a new one-trial protocol called Inhibitory Avoidance Task (IAT).In the 1960’s, Jarvik and Kopp[4] modified the IAT by building a novel apparatus containing 2 compartments, a small lit chamber connected to a larger dark chamber, where the animal received a shock as it entered to the dark compartment. Thereafter, the protocol for IAT has been modified several times. These changes resulted in a well-established protocol of Step-down inhibitory (passive) avoidance task[5–16]

In step-down inhibitory avoidance task, one critical point was the application of various electric current intensities, ranging from 0.3mA to 3mA[17–20].The stimulus intensities reflect in the latency of step-down in the test trial (24 h after training). For male Wistar rats (age, 2–3 months, weight, 220–260 g), researchers showed the latency for step-down in the test of ±40–50 seconds with 2 seconds of stimulus in intensities of 0.3mA [7],0.4mA[15,21] and 0.5mA[22–26];step-down latencies of 180s in test applied 0.8mA (2s) in training[22,27] and 600 s for 1mA (2s)[28].

However, these apparatus did not consider the influence of animal’s bioimpedance on the applied currents. Additionally, several other versions of protocol combine one or two-trials assessments during the task[21].Therefore, up to now there is no standardized protocol for IAT to properly evaluate aversive memory [29], principally for fresh researchers in the field.

Therefore our aim was to develop and standardize a new device for step-down inhibitory avoidance task, in which the applied electric current has the precise same intensity as the current passing through the animal paws, taking in consideration the animal bioimpedance; the stimulus frequency and intensity are controlled and recorded during the entire experiment. For further tasks, we challenged this new step-down inhibitory avoidance device using a known amnesic drug (guanosine,i.p.) [30,31].

## Materials and Methods

### Animals and experimental conditions

All experimental procedures were performed according to the Brazilian Law N° 11.794/2008. Recommendations for animal care were followed throughout all the experiments in accordance with project approved by the ethical committee from the Federal University of Rio Grande do Sul-UFRGS #5647. Adult Wistar female (3 months old, 280–300g, n = 20) and male (3–5 months old, 310–450g, n = 258) rats were used in the present study. Animals were allocated in a controlled temperature (22°C ± 1°C) room under a 12h light/12h dark cycle. They were distributed, in groups of 5 in Plexiglas cages with free access to food and water. Previously to the experiment the animals were handled using gloves, for approximately 5 min, once a day for 7 consecutive days. This step is critical to habituate the animals to the experimenter and reduce the stress caused by manipulation. After the habituation period the animals were evaluated in the inhibitory avoidance apparatus between 3:00 to 5:00 PM. The animal behavioral performance was video recorded.

Two distinct protocol paradigms were used: in the first, the duration of the foot shocks was set in 3s[26,30,32] adapting the frequency and current applied; in the second, animals receive foot shock until they return to the platform[20]. The time parameters, including the step-down latency to the grid with the 4 paws during the training and the test sessions were accessed with a manual activated chronometer.

### Inhibitory avoidance apparatus

**Fig 1:**
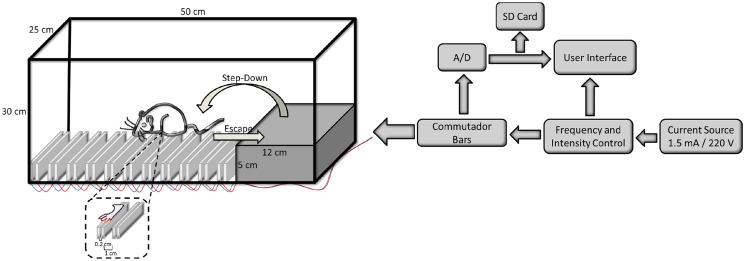
Schematic representation of the inhibitory avoidance apparatus. The inhibitory avoidance apparatus, including operating diagram with digital frequency control by microcontroller (PIC18F4520), an analog-digital (A/D) to measure and to control the intensity of stimulus and a data recorder on a SD card.

The inhibitory avoidance apparatus consists of a 50 × 25 × 30 cm poly(methyl methacrylate) box. This box has a 48 × 30 cm transparent acrylic window, a 5 cm-high,12 cm-wide and 25 cm-long platform on the left facing a grid of a series of 20 pairs of stain steel bars (2mm diameter) spaced 2mm within bars and each pair spaced 1 cm apart (Fig. 1). Each pair of bars makes an electrical dipole where the polarity inverted after each pulse. All bars have insulating layer on the sides and bottom. This step-down inhibitory avoidance apparatus was built to avoid the variations of current intensity received by animals caused by the animal bioimpedance. With a new arrangement and bars design the electrical current pass through only the animals’ paws that is in contact with each pair of bars. This allows us to control the current frequency and intensity and to record the current passing through the paw of the animal during the entire experiment.

**Fig 2:**
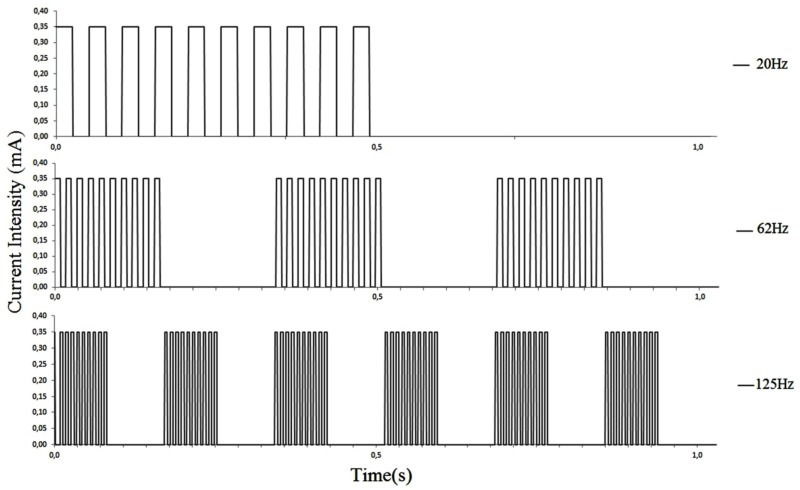
Positive phase of stimulus waveform for different frequencies (Hz) at 0.35mA current intensity.

**Frequencies**. Three different frequencies of current were applied, as shown in the Fig. 2: trains of pulses at 20Hz (10 pulses of 25ms every 500ms), 62Hz (10 pulses of 8ms every 167ms) and 125Hz (10 pulses of 4ms every 83ms). Thus, the period of shock applied in all selected frequency, in one second, was the same. Programing the current frequencies, we introduce one modulate frequency, which exposes the animal to pulse train followed by periods without shock, it avoid the continuous animal muscle contraction provoked by passing of electric current and allows the animal to escape.

The choice of frequency was based in frequencies utilized in electric stimulation machines for physiotherapy proposes using 10–100Hz[33]. The frequency of 10Hz was used by Garin[20], and the value of frequency in major articles used the inhibitory avoidance apparatus wasn’t specified.

These frequencies are controlled by a microcontroller PIC18F4520. The intensity of stimuli varied according to the protocol used and it is specified for voltage received by 100ohms resistor integrated in IAT circuit. This voltage was converted in a A/D port of microcontroller PIC18F4520 with 10bits resolution, in accordance with Nyquist-Shannon sampling theorem, converted in a ASCII number and recorded in a. txt file in a SD card (2Gb) using the fat16 library for C++.

**Bioimpedance**. Important aspects have to be considered when using the inhibitory avoidance apparatus. The voltage source is normally 110 or 220V and the resistance changes by a selector switch, the application of the direct current provided by batteries is used, however it result in an higher influence of bioimpedance of the animal [34]. According the Ohm’s law, the bioimpedance decreases the effective current received by the animal when it steps on the grid[35]. There are three measures involved in bioimpedance value (using a frequency current below 1kHz): the conductivity value of animal’s body (ρ), the contact area between animal’s foot and iron bars (A) and the distance between bars (L). In frequencies below 1kHz the bioimpedance is equal as the body resistance R = ρ.L/A [36]. We substantially increase the area (from 0.2cm² to 0.8cm²) modified the bars shape and decrease the distance (from ~15cm to 0,2cm) between ground and positive poles. As a result of our modifications we present a new device with a large (A) and small (L) resulting in a drastically decrease in the bioimpedance values of paw skin [37] from 1.7 Momhs to 32 Komhs.

**Fig 3:**
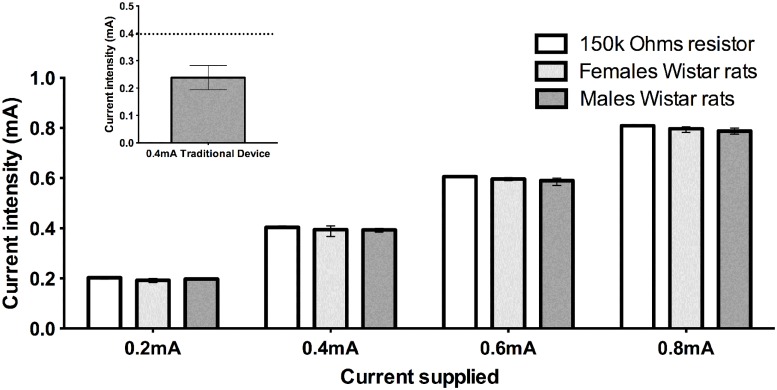
Plots of current intensity at 62Hz passing through the animal’s paws or resistor. Different groups were represented by white, light gray or dark gray bars: 150Kohms resistor (no animal in the apparatus), female rats (3 months; 280–320g) or male rats (3 months; 310–360g), respectively. In insert graphic the effective current passing by animal body in traditional apparatus of inhibitory avoidance, nominal current 0.4mA. The standard data represents mean±S.D. Analyses by Mann-Whitney test; n = 5 animals/group/current supplied and 5 measurements for each animal. No significant difference was observed among the groups. Insert graphic, 8 animals, 3 measurements for each animal.

In the traditional apparatus the skin bioimpedance of 1.7 Mohms/1mm^2^ is very similar to the internal resistance of a current source of 1mA[37] and the bioimpedance of animal changes with body composition[38].This aspect has not been specified by many authors[3,20,30,39,40]. Indeed, we measured the value of current intensity in the traditional apparatus of Inhibitory Avoidance Task, with a floor containing parallel caliber stainless-steel bars (1mm diameter) spaced 1 cm apart [41,42]. So the effective current received by the animals´ paws in traditional apparatus usually is reduced by 21–58% of the selected value when the paws touch the bars (Fig. 3, insert).In simple words, a researcher selecting 0.5 mA in the traditional apparatus can be applying 0.25–0.4 mA, but not the choice intensity presenting a high variation in the final results.

In our Inhibitory Avoidance apparatus, we increased the contact area of the paws skin and the pair of bars by 0.8 cm² and we decrease the current route, consequently, we drastically decreased the impedance values of paw skin [37] from 1.7 Momhs to 32Komhs. By performing these changes we were able to reduce the decrement of effective current from 21–58% (traditional apparatus) to 0.1–3%.

To the electrical shock pass only in the paws and not through the body, in our apparatus, there are three position modes of bars: connected in phase, connected in ground, and off (no phase, no ground). We turn on the pars of bars, one by one, connected only one bar in phase and only one bar in ground each time, all others bars was off. The bars of pair were close enough for the animal touch, with one foot, the two bars at the same time (positive and ground bars), so the current pass from the positive bar to the ground bar by the pawn of the animal and not by the body as the traditional apparatus (Gauss Law).

**Recording the current**. It should be observed that, besides bioimpedance, electrical conduction also depends on “extra-corporeal factors” such as any dirt interposed between the paws and the bars during tests (faeces, etc.), another factor hard to quantify. This is the reason for measurement and record the current passing in the animal, if any extra-corporeal factors decrease the current intensity it will be visualized.

The current was recorded by an analog/digital converter (A/D) and the sampling rate followed the Nyquist-Shannon theorem. The sampling frequency of 400 Hz was used. We measured the current value by the voltage in a 100ohms resistor placed after the negative pole bar, so the current intensity value was registered only when the animal´s paw closed the circuit stepping on a pair of bars. The value measured (*V*_2_) is the resultant of the voltage applied (*V*_1_) divided by the summation of resistance in the switch (*R*_1_), the bioimpedance of animal´s paws (*R*_2_) and the 100ohms resistor (*R*_3_), multiplied by 100ohms (see. 1).

$$V_{2}=\frac{R_{3}V_{1}}{\left(R_{1}+R_{2}+R_{3}\right)}$$ (1)

This value is then converted to ASCII character by the microcontroller and record on a SD Card.

**Fig 4:**
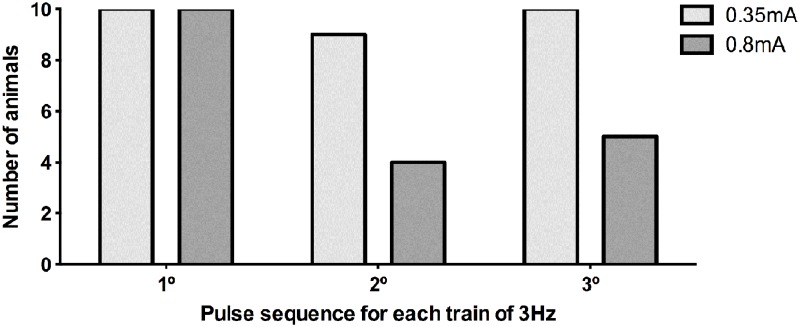
Number of animals (n = 10 per group) receiving 3 consecutive bursts of current (0.35 or 0.8mA, 62 Hz), measured for 1 s.

Using this recording system, we were able to determine the number of stimuli received by each animal. The Fig. 4 shows the data of stimuli intensity of 0.35 and 0.8mA at trains of 62Hz.When the stimulus intensity were of 0.35 mA at trains of 62Hz or 125Hz, all animals received the exact given pulses, with no jumping. However, with stimulus intensity of 0.8mA, the number of jumps increased and consequently the exact given pulses received by the animals decreased, jeopardizing the experimental analysis. Therefore, to avoid the inconsistent amount of received stimuli due to the jumping, we decided to use the stimuli intensity of 0.2, 0.35 and 0.5 mA to evaluate the effect of different current frequencies on the performance of the animals (all data of the current intensity passing through the animal paws were recorded in a SD Card and analyzed offline).

### Step-down Passive Inhibitory Avoidance Task

**First protocol**. For this protocol, we used the stimulus of 0.35mA at 62Hz. Each animal was gently placed on the platform facing the rear left corner of the inhibitory avoidance apparatus (training session).When the animal stepped-down on the grid with the 4 paws, it received the footshock (stimulus) for 3 seconds. Afterwards, the animal was withdrawn from the apparatus. After 24h (test session), each animal was placed on the platform again. In this session no shock was applied when the animal stepped down on the grid. After stepping down, each animal was withdrawn from the apparatus and placed back to the home cage [43]. The time parameters were accessed during the training and the test sessions with a manual activated chronometer. The time to step down on the grid with the 4 paws was measured in both, training and test sessions. Step-down latencies were cut-off at 60s in the training session and at 180s in the test session.

**Second protocol**. During the training session, each animal was gently placed on the platform facing the rear left corner of the inhibitory avoidance apparatus. Immediately after stepping down with the 4paws on the grid the animal received continuous scrambled footshock (the current and frequency depended on the specific group) until it spontaneously step-up back to the platform (latency to escape). The animal was then withdrawn from the apparatus. The escape latencies were cut-off at 60s[20].After 24h (test session), the procedure was performed as described for the test session of first protocol. These time parameters were accessed during the training and the test sessions with a manual activated chronometer. Step-down latencies were cut-off at 60s in the training session and at 180s in the test session. The animals were divided at 11 groups according to the intensity and frequency stimuli applied (0.2mA; 20,62 and 125Hz), (0.35mA; 10,20,40,62 and 125Hz), and (0.5 mA; 20, 62 and 125 Hz).

**Passive inhibitory avoidance performance (Score)**. To evaluate the performance in IAT, the latency to step-down in the training and test sessions are measured. According to previous studies, the memory was measured by the difference between the latency to step-down during the test session and the latency to step-down in the training session [30–32,43,44][22–26].In second protocol, the time to scape change for each animal, so we qualified the performance by the influence of the time to explore the platform (step-down latency in training session), the foot shock duration (latency to escape) and the latency for step-down in test (step-down latency in test)to evaluate the inhibitory avoidance performance(see 2).

$$f(t)=\frac{log(k_{1}/SDT)}{\left(k_{2}.SU\right)}.SD24H$$ (2)

*f(t)*: IAT function

*SDT*: step-down latency in training session

*SD24*:step-down latency in test

*SU*: latency to escape

*k*_1_: 60s (maximum time in the platform during the training session)

*k*_2_: 3s (minimum time in the platform during the training session)

The measure of “learning” was the same as all classic articles using the passive inhibitory avoidance task, so we measured the difference between step-down latency in train and the step-down latency in test after 24h.The value of latency to return to platform was used to qualify and compere animals with different time to return to platform in function of the leaning in task.

Using the equation *f(t)* we can compare the effect if a treatment that affects the latency to return to platform (ex: sensibility to the shock),do or do not affects learning. If the time to return to the platform is different among the animals, the equation balances this variable resulting in a value of performance.

Additionally any values of equation above 1 indicate the step-down latency in test (*SD24H*) is significant higher of step-down latency in training (*SDT*), independent of the escape latency.

### Drugs

Guanosine (GUO) from Sigma (St. Louis, MO, USA) was dissolved in NaCl 0.9%. The solutions were prepared immediately before use and were protected from the light[30,45].Thirty minutes before the training session the rats received a single intraperitoneal (i.p.) injection of 7.5mg/kg GUO (1ml/kg)or vehicle (NaCl—0.9%). The dose of GUO applied was based on previous studies from our group using *in vivo* protocols to investigate the anticonvulsant [46–49] and the amnesic effect on IAT [30].

### Statistical analysis

The data are expressed as median and interquartile range, since the behavioral variables being analyzed (step- down and step up- latency) did not follow a normal distribution, using D’Agostino&Pearson omnibus test of normality. The observation was limited to 180 seconds. The data were analyzed using the Graphpad Prism 5 program. To compare the results we used One-Way ANOVA followed by Dunnett’s, Mann-Whitney, or using Chi-square with significance level of *p*< 0.05.

**Fig 5:**
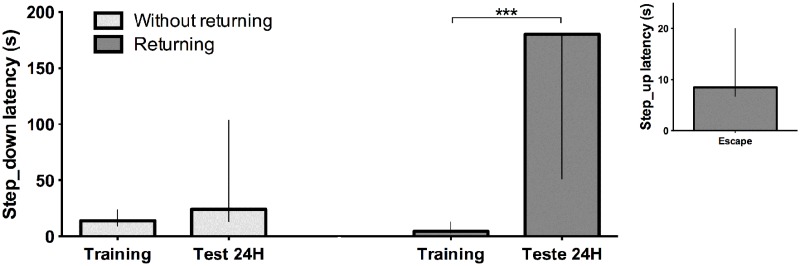
Step down latency (s) for training and test sessions. Light gray represents the group without returning (first protocol with 3s of stimulus), and dark gray represents the group returning (second protocol with escape latency) in the training session (0.35mA, 62Hz). The latency to escape in the second group is represented in the insert graphic. The data is expressed by median with interquartile range; analyses by Mann-Whitney test; (n = 10 per group) ***p<0.001 comparing to SDT.

**Fig 6:**
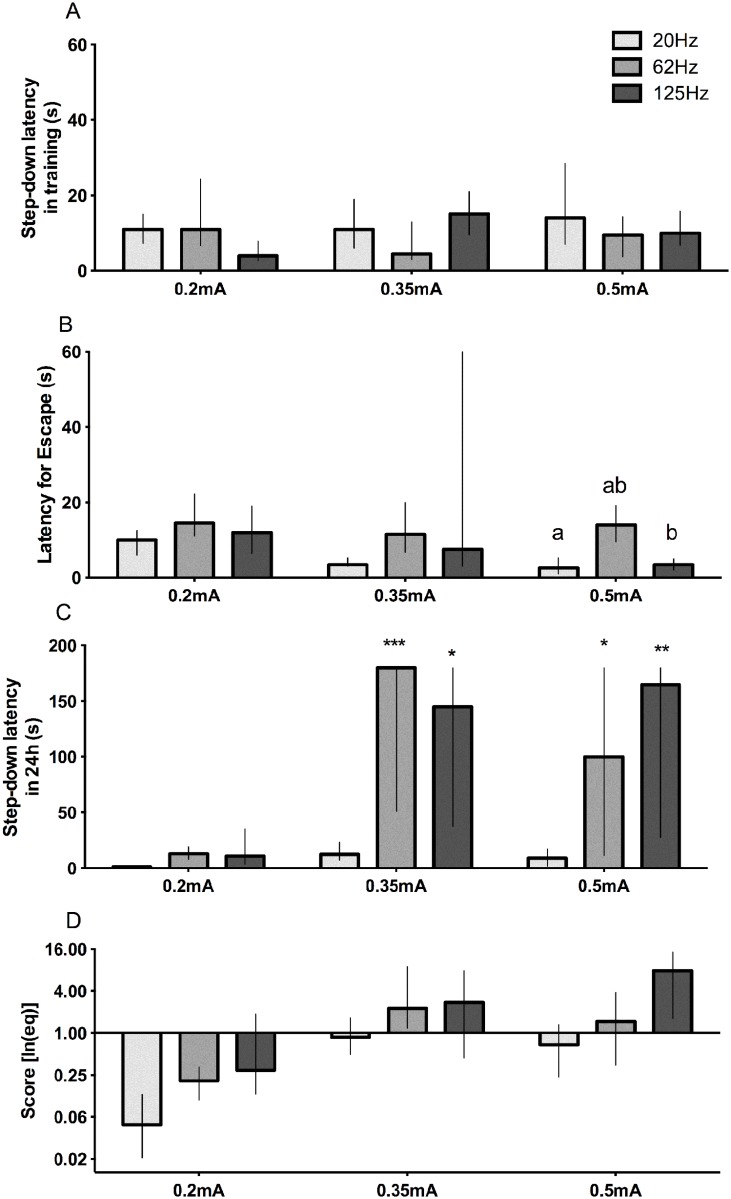
Effects of frequency (Hz) and current intensity (mA) in step-down inhibitory avoidance parameters. Effects of frequency (Hz) and current intensity (mA) on step down latency in training session—A, latency for escape—B, step down latency in test session—C, and score performance (D—the combination of the 3 graphs above by applying the Equation 2). In B, same letter represent statistical difference among groups; *p<0.05;**P<0.01;***p<0.001, by Mann-Whitney, comparing to the same group in A. Data represent median with interquartile range (n = 10/group).

**Fig 7:**
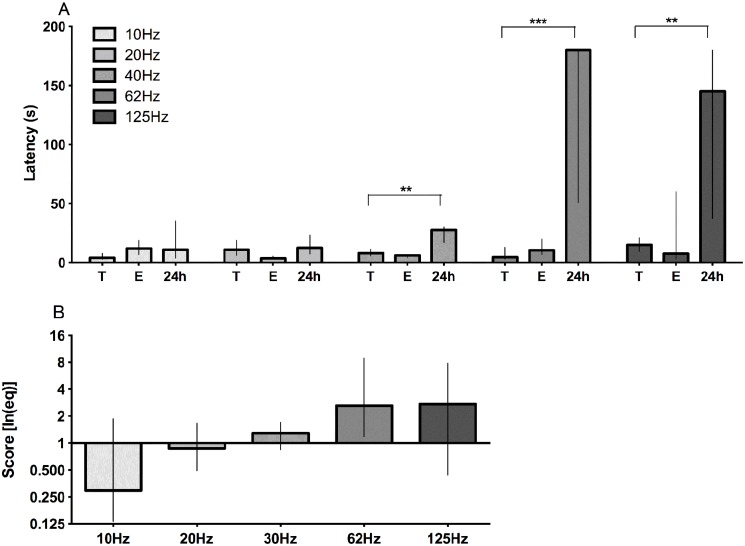
Effects of frequency (Hz) with 0.35mA current intensity in step-down inhibitory avoidance latencies. A- Effects of frequency (Hz) on step down latency in training session (T), latency for escape (E), step down latency in test session (24h), and score performance (B—the combination of the 3 parameters by applying the Equation 2).**P<0.01; ***p<0.001, by Mann-Whitney, comparing to the same group in A. Data represent median with interquartile range (n = 10/group).

**Fig 8:**
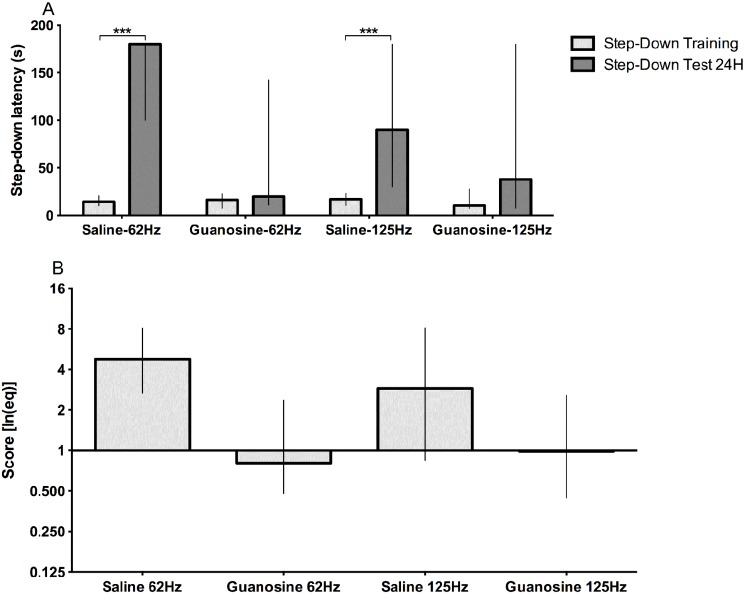
Effect of guanosine on the step-down inhibitory avoidance task. (A) Effect of guanosine (i.p.- 7.5mg/kg) on the step-down inhibitory avoidance task using 0.35 mA stimuli with different frequencies (62 Hz and 125 Hz). All groups displayed similar escape latency (data not shown). Bars express median (interquartile range). (B) Application of Equation 2 to the data of the graph A. The data were analyzed by Mann-Whitney test (n = 20 per group); ***p<0.001.

Regarding total *n* = 278 we can divide in: current intensity measurement—48 animals (Fig. 3); measurement of consecutive bursts of current—20 animals (Fig. 4); protocol 1 and protocol 2 comparison–20 animals (Fig. 5); influence of current and intensity measurement—90 animals (Fig. 6); mapping of 0.35 mA effects—20 animals (Fig. 7); guanosine experiment—80 animals (Fig. 8).

## Results

Using our new step-down inhibitory avoidance device, the current received by the animal through the paws was exactly the same current applied without any interference of the body bioimpedance for all stimuli intensities (0.2, 0.4, 0.6 or 0.8 mA) (Fig. 3). However, to obtain this result, it is necessary to turn on one pair of bars at a time. This is controlled by the microcontroller PIC18F4520that witches the pair of bars according the selected train of frequencies (20Hz, 62Hz or 125Hz) and the number of pair (20 pair in this apparatus).

In the second protocol (variable escape time), the median time that animals received shock was of 8.5s (interquartile range, 7.25–18.5 seconds) and the animals spent more time in the platform during the test session compared to the training session (Fig. 5). The learning was observed in the second protocol but not in the first protocol (applied the stimulus for 3s), using 0.35mA 62Hz.However, using current of 0.8mA 62Hz for 3s the animal behavior reflects the classic experiments (data not show). Therefore, we choose to use the second protocol for further experiments in this study.

There was no statistical difference among groups in the step-down period during the training session (Fig. 6A).There was statistical difference among groups 0.5mA 62Hz and 0.5mA 20–125Hz in the latency to escape from the grid (Fig. 6B). Moreover, for the stimuli intensity of 0.2mA at 20, 62 or 125Hz, and 0.35 or 0.5 mA at 20Hz there was no difference in step-down latency in test session when compared to the respective step-down latency in training (Fig. 6C). Animals receiving stimuli intensity of 0.35 and 0.5 mA at 62 and 125Hz had increased time spent in the platform in test session (Fig. 6C). Applying the Equation 2 we can observe the influence of all variables evaluated in this study that may contribute for the learning process. By these mathematical analyses all groups with a median above 1 are classified as presenting memory (Fig. 6D).

The Fig. 7 shows the effect of 0.35mA stimulus with different frequencies in step-down latency in test session. For 40Hz the step-down latency in test session is similar to observed when used 0.3–0.5mA in classic articles (median of 40–50 seconds), however, when we use 62Hz the step-down latency is comparable to 0.8mA (median of 180 seconds), and the behavioral response to the stimulus is more intense at higher frequencies (Table 1).

**Table 1:** Description of the observed behavioral responsesduring the test and training sessions.

**0.35mA 20Hz**								
**Training**				**Test**				
**ID**	Vocalization	Flinch	Jump	Rearing	Turn Angle	Freez	Risk Acessment	Faeces
**cx1 r1**	-	+	-	2	0	0	0	0
**cx1 r2**	+	+	-	6	6	1	2	Ok
**cx1 r3**	-	+	-	3	2	0	2	Ok
**cx2 r3**	-	++	-	5	3	0	2	0
**cx2 r4**	+	+	-	2	1	0	1	Ok
**cx3 r4**	+	++	-	1	0	0	0	Ok

**0.35mA 62Hz**								
**Training**				**Test**				
**ID**	Vocalization	Flinch	Jump	Rearing	Turn Angle	Freez	Risk Acessment	Faeces
**cx1 r4**	++	++	-	10	4	0	7	Ok
**cx1 r5**	+++	+++	+	5	5	0	5	Ok
**cx2 r1**	++	++	+	7	3	0	6	Ok
**cx2 r2**	++	++	+	3	2	0	5	0
**cx3 r1**	+++	+++	-	5	3	0	6	Ok
**cx3 r2**	+	+++	+	14	8	0	7	Ok
**cx3 r3**	+++	+++	+	2	1	0	3	Ok

+ one episode,

++ between two and four episodes,

+++ more than four episodes; in test the absolute number during the session.

**Fig 9:**
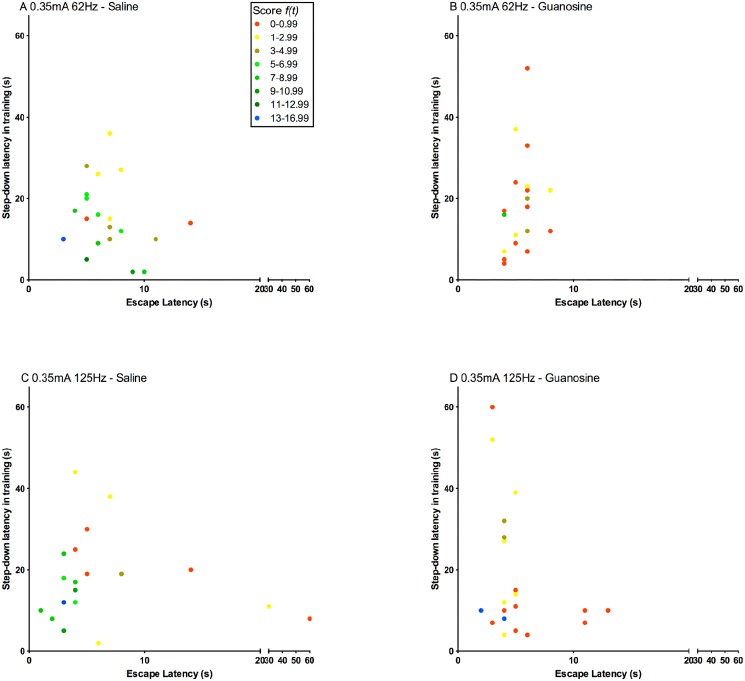
Platform exploration time in training (step-down latency in training) and time of stimulus (escape latency) influence on learning. The scale of colors indicates the animals score based in Equation 2. (A) Saline group exposed at 0.35mA, 62Hz. (B) Guanosine group exposed at 0.35mA, 62Hz. (C) Saline group exposed at 0.35mA, 125Hz. (D) Guanosine group exposed at 0.35mA, 125Hz. (n = 20/group).

Using the second protocol we observed diminishing of memory in avoidance task by guanosine at i.p. 7.5mg/kg at 0.35mA, at 62Hzand 125 Hz stimulation (Fig. 8), even though the guanosine group presented different time to return to the platform among the animals of the group, there were still the amnesic effects of this compound. There was influence of latency to return to platform in test (escape latency) and the step-down latency in training on the performance score in both, saline (Fig. 9 A-C) and guanosine (Fig. 9 B-D) administration. We observed that 18% of animals treated with GUO and 17.5% of control group obtained a score ≥1 and < 2.99. However, the number of animals treated with GUO with score higher than 3 where significantly smaller than the control group (17%, 65%, respectively) (p<0.0001, Chi-square) (see Fig. 9).

## Discussion

In our new apparatus, the electric stimulus does not depend of animal’s bioimpedance (Fig. 3). It permits to evaluate the real effect of the stimulus among groups with different bioimpedance values, eliminate the influence of house conditions[50]the phenotype, genetic[34,35], treatments like exercise [51] and drugs[52], changing the bioimpedance value[53].

Further, our aim was to design an apparatus with stimulus frequency and intensity easily and clearly controlled and recorded during the entire experiment, as the studies using traditional apparatus designed to test step-down avoidance task do not evaluate the influence of animal’s bioimpedance on the applied currents, nor frequency, neither the intensity of stimulus. These 3 points clearly alter the behavior of the animals, as confirmed by our results.

Among the advantages from these modifications we can sustain that the current applied during the task is the current arrived to the animals. In simple words, a researcher selecting 0.5 mA in the traditional apparatus can be applying 0.25–0.4 mA, which result in a high variation in the final performance of the animals. With this new apparatus, we were able to reduce the decrement of effective current from 21–58% (traditional apparatus) to 0.1–3% (Fig. 3). Using the traditional apparatus for step-down inhibitory avoidance task, each animal may receive different electric current intensity (due variations in body composition); so the comparison among animals with different ages, sex or weight is jeopardized, due to the animals bioimpedance differences.

It should be observed that, besides bioimpedance, electrical conduction also depends on “extra-corporeal factors” such as any dirt interposed between the paws and the bars during tasks (faeces, etc.), another factor hard to quantify. This is an additional reason for measurement and recording of the current arriving to the animals, if any extra-corporeal factors decrease the current intensity it will be visualized.

The frequency modified the effect of current intensity on leaning in inhibitory avoidance task and the behavioral of the animal during the stimulus (Table 1). It’s a nonstandard parameter and usually not cited in articles, but it is important as the current intensity confirmed by our data (Figs. 6 and 7).

The choice of frequency was based in frequencies utilized in electric stimulation machines for physiotherapy proposes using 10–100Hz[33]. The frequency of 10Hz was used by Garin[20], and the value of frequency in most articles used the inhibitory avoidance apparatus wasn’t specified. Even using a battery as electric source, the stimulus should be given in form of pulses, the constant current (Gaussian current) promote fixed muscle contraction and can burn the skin (Joule effect). Moreover we need to have some kind of ideal frequency window to work. In this sense, we worked with a low frequency as 20 Hz to indirectly answer what could be the lesser frequency for the learning in this task.

Here, there was a relation between performance in step-down inhibitory avoidance task and the current frequency (Figs. 6 and 7). The behavioral escape, but not the performance in test session, was the same for 20 Hz and 125 Hz frequencies. This modified step-down inhibitory avoidance apparatus allowed us to control variables that have been not specified up to now [18,32,39,54]. Our data demonstrates that not only the intensity but also the frequency of the applied current plays a key role in the performance in a step-down inhibitory avoidance and learning. This data of frequency is one of missing information in other studies using IAT [19,41,55,56]. We observed learning with 0.35mA and 0.5mA at 62 and 125Hz but not at 20Hz (Fig. 6). The latency to step up back to the platform during the training session was similar for all current intensities, even for the lowest applied current (0.2mA), regardless the frequency applied.

It is important to emphasize that the possibility to programing the current frequencies in our apparatus with one modulate frequency (Fig. 2) allows to avoid the continuous animal muscle contraction provoked by the passing of electricity by the animal body. Also, the shape of bar avoid that animal hold the bar during the stimulus (Fig. 1). In order to maintain this concept we needed to resolve the problem of bioimpedance influence in electric current. Thus, we changed the bars distribution, shape and turn on mode. In this way we cannot apply a scrambled footshock in this model, as the classical apparatus, because we need to turn off the bars. Furthermore, a comparison among traditional systems and the ours (with and without influence of bioimpedance, respectively), is difficult because we cannot control the frequency in traditional apparatus, nor to measure the real intensity through the animals

With the new apparatus presented here we were able:(1) to abolish the influence of bioimpedance in the intensity of the current received by the animal; (2) to precisely control the current received during the shocks delivery over the task; (3) to control and constantly record the intensity and frequency of the effective current applied, enabling a full record of the entire experiment. There was a clear reduction in the decrement of effective current to only 0.1–3%, indicating an insignificant interference of bioimpedance (Fig. 3).

The guanosine, a guanine-based purine, plays important roles in the Central Nervous System[49,57], and has a well establish amnesic effect *in vivo* in rodents[30,31,49]. Therefore we evaluated our new device using animals treated with i.p. injection of guanosine. We observed a significant diminishing effect of guanosine on step-down inhibitory avoidance memory even with a variable escape time.

Taken altogether, this new device offers a substantial improvement in behavioral analysis in the step-down inhibitory avoidance task, by considering crucial parameters that havebeen never stated before, such as frequency of stimulus application and principally the influence of the animal’s bioimpedance. This influence was drastically reduced by the changes in the shape and distribution of the bars in the apparatus, which permitted to record the exact current passing precisely trough the paws of the animal during the task and to compare results among animals with different body composition. Despite all these findings, only future studies with classical impaired memory diseases or potential memory modulators drugs could affirm the full potential of our new apparatus. Finally, we also propose a mathematical equation that could improve the analysis of animal performance in the inhibitory avoidance task.
